# Defective Autophagy in Vascular Smooth Muscle Cells Alters Vascular Reactivity of the Mouse Femoral Artery

**DOI:** 10.3389/fphys.2020.548943

**Published:** 2020-09-23

**Authors:** Dorien G. De Munck, Sofie De Moudt, Lynn Roth, Guido R. Y. De Meyer, Wim Martinet, Paul Fransen

**Affiliations:** Laboratory of Physiopharmacology, Department of Pharmaceutical Sciences, University of Antwerp, Antwerp, Belgium

**Keywords:** autophagy, vascular smooth muscle cell, femoral artery, contractility, angiotensin II

## Abstract

Autophagy is an important cellular survival process that enables degradation and recycling of defective organelles and proteins to maintain cellular homeostasis. Hence, defective autophagy plays a role in many age-associated diseases, such as atherosclerosis, arterial stiffening and hypertension. Recently, we showed in mice that autophagy in vascular smooth muscle cells (VSMCs) of large elastic arteries such as the aorta is important for Ca^2+^ mobilization and vascular reactivity. Whether autophagy plays a role in the smaller muscular arteries, such as the femoral artery, and thereby contributes to for example, blood pressure regulation is currently unknown. Therefore, we determined vascular reactivity of femoral artery segments of mice containing a VSMC specific deletion of the essential autophagy gene Atg7 (Atg7^F/F^ SM22α-Cre^+^) and compared them to femoral artery segments of corresponding control mice (Atg7^+/+^ SM22α-Cre^+^). Our results indicate that similar to the aorta, femoral artery segments showed enhanced contractility. Specifically, femoral artery segments of Atg7^F/F^ SM22α-Cre^+^ mice showed an increase in phasic phenylephrine (PE) induced contractions, together with an enhanced sensitivity to depolarization induced contractions. In addition, and importantly, VSMC sensitivity to exogenous nitric oxide (NO) was significantly increased in femoral artery segments of Atg7^F/F^ SM22α-Cre^+^ mice. Notwithstanding the fact that small artery contractility is a significant pathophysiological determinant for the development of hypertension, 7 days of treatment with angiotensin II (AngII), which increased systolic blood pressure in control mice, was ineffective in Atg7^F/F^ SM22α-Cre^+^ mice. It is likely that this was due to the increased sensitivity of VSMCs to NO in the femoral artery, although changes in the heart upon AngII treatment were also present, which could also be (partially) accountable for the lack of an AngII-induced rise in blood pressure in Atg7^F/F^ SM22α-Cre^+^ mice. Overall, our study indicates that apart from previously shown effects on large elastic arteries, VSMC autophagy also plays a pivotal role in the regulation of the contractile and relaxing properties of the smaller muscular arteries. This may suggest a role for autophagy in vascular pathologies, such as hypertension and arterial stiffness.

## Introduction

Cardiovascular diseases (CVDs) are the leading cause of death and morbidity worldwide. According to the World Health organization (WHO), an estimated 17.5 million people die annually from CVDs (Benjamin et al., 2017). Although coronary heart disease is the most common cause of death, hypertension is also a major health concern because it is an important risk factor not only for atherosclerosis, but also for myocardial infarction, stroke, aortic aneurism formation, hypertensive heart disease, heart failure and even renal injury. Due to highly effective therapeutic interventions, the lifespan and wellbeing of patients with CVDs has improved significantly, albeit a large group of patients still suffer from complications due to CVDs (Benjamin et al., 2018). Therefore, additional treatments are needed.

Autophagy is considered an important life-sustaining process, which is involved in multiple *in vivo* processes. A deficiency in autophagy is associated with age related diseases (Ren and Zhang, 2018; De Munck et al., 2020a). Previously, we showed that autophagy in vascular smooth muscle cells (VSMCs) is involved in large artery contractility and stiffness (Michiels et al., 2015; De Munck et al., 2020b). Interestingly, epidemiological studies show that arterial stiffness precedes the development of hypertension (Najjar et al., 2008; Kaess et al., 2012). However, they are both strongly interconnected, as positive feedback systems exist in which they progressively aggravate each other (Humphrey et al., 2016; Safar et al., 2018). In addition, vascular segments of spontaneous hypertensive rats and aged mice show a decrease in autophagy markers (McCarthy et al., 2015). Although several autophagy inducers, such as spermidine and resveratrol reduce the development of hypertension, a direct link between autophagy and essential hypertension is still lacking (Dolinsky et al., 2013; Eisenberg et al., 2016, 2017).

We previously demonstrated that VSMC autophagy affects the contractility of the thoracic aorta (Michiels et al., 2015). However, blood pressure is mainly defined by the resistance vessels rather than the aorta. As elastic and muscular arteries differ in structure, basal NO production and voltage-gated Ca-channels (Leloup et al., 2015), it is important to decipher the implications of defective autophagy in VSMCs of both large elastic arteries (such as the aorta) and smaller muscular arteries. For this reason, in the present study, we investigated the effects of an autophagy defect in VSMCs on the vascular reactivity of the femoral artery. We also aimed to clarify the influence of autophagy on blood pressure and the development of hypertension.

## Materials and Methods

### Mice

Mice on a C57BL/6 background with a selective Atg7 gene deletion in VSMCs (Atg7^F/F^ SM22α-Cre^+^) (Michiels et al., 2015) and wild-type littermates without the Atg7 deletion but expressing SM22α-Cre (Atg7^+/+^ SM22α-Cre^+^) were housed in the animal facility of the University of Antwerp in standard cages with a 12:12 h light-dark cycle and had access to regular chow and water *ad libitum*. At the age of 2 months, animals [both male (*n* = 15) and female (*n* = 3) mice] were euthanized by perforating the diaphragm after anesthesia with pentobarbital sodium (Sanofi, 250 mg kg-1, i.p.). The femoral artery was carefully isolated systematically and stripped from adherent tissue. Atg7^F/F^ SM22α-Cre^+^ mice and Atg7^+/+^ SM22α-Cre^+^ mice were dissected in parallel. Vessel segments were immersed in Krebs Ringer (KR) solution containing (in mM) 118 NaCl, 4.7 KCl, 2.5 CaCl_2_, 1.2 KH_2_PO_4_, 1.2 MgSO_4_, 25 NaHCO_3_, 0.025 CaEDTA and 11.1 glucose; pH 7.4 at 37°C and continuously aerated with 95% O2/5% CO_2_. High K^+^ solutions were prepared by replacing NaCl with equimolar KCl. 0Ca^2+^ solution was prepared by omitting Ca^2+^ from the KR solution and adding 1 mM EGTA (Sigma-Aldrich) to chelate remaining Ca^2+^ residues. All animal procedures were approved by the ethics committee of the University of Antwerp.

### Isometric Experiments

Femoral artery vessel segments (2 mm width) were mounted in a wire myograph (DMT, Denmark). After a short equilibration period of 30 min, the segments were gradually stretched until wall stress attained values above 13.3 kPa (100 mmHg). Segments were then set at the internal circumference according to 90% of the 13.3 kPa stress, after which all transducers were re-set to zero tension in order to measure active tension. Contractile tension was measured and reported in mN/mm. Contractions of vessel segments were elicited by increasing concentrations of KCl (K^+^: 5.9, 10, 15, 20, 25, 30, 35, 40, 50 mM) (VWR). Transient IP_3_ mediated contractions were induced by phenylephrine (PE, 2 × 10^–6^M) (Sigma-Aldrich) after segments were incubated for 3 min in a Ca^2+^ free environment (0Ca^2+^). To restore normal conditions hereafter, 3.5 mM Ca^2+^ was added to the 0Ca^2+^ solution. Subsequently, relaxation was induced with increasing concentrations of acetylcholine (ACh, 3 × 10^–9^ – 10^–5^ M) (Sigma-Aldrich) or with the NO donor Diethylamine NO-NOate (DEANO, 3 × 10^–10^ – 10^–5^ M) (Sigma-Aldrich). Absolute values of contraction and relaxation were normalized to the maximal (pre-)contraction for the calculation of respective relative contraction and relaxation curves. A representative example of the experimental protocol applied to the femoral artery segments is shown in a Supplementary Figure 1. Vessel segment VSMC viability was assessed by analyzing the contractile and relaxing responses, whereas endothelial cell (EC) function was evaluated via acetylcholine-induced relaxation.

### Angiotensin II Treatment

At 6 weeks of age, Atg7^F/F^ SM22α-Cre^+^ and Atg7^+/+^ SM22α-Cre^+^ mice were anesthetized (sevoflurane 4–5% v/v, SevoFlo, Penlon vaporizer) and subcutaneously implanted with osmotic minipumps (model 1007D, Alzet) filled with angiotensin II (AngII) (1000 ng/kg/min) or PBS as a control. The minipumps allowed continuous administration of AngII over a period of 7 days. At day 7 of AngII treatment mice were euthanized.

### Blood Pressure Measurements

To measure peripheral blood pressure, we were unable to use telemetric methods. Especially at this young age, implanting telemetry catheters was associated with a high mortality. In addition, the combination of telemetric methods with echocardiography is complicated by insertion of the telemetric probes. Therefore, peripheral blood pressure was measured by a CODA tail-cuff method as previously described (Fransen et al., 2016). In brief, conscious, restrained mice were held in a heating chamber (37°C) for 10–15 min to acclimatize before measurements were initiated. A pneumatic pulse sensor was attached to the tail and controlled distal to an occluding cuff by a programmed electro-sphygmomanometer (Narco Bio-Systems, Austin, TX, United States). Voltage output from the cuff and the pulse sensor was recorded and analyzed by a PowerLab signal transduction unit and associated Chart software (ADInstruments, Colorado Springs, CO, United States). At least four sessions of recorded measurements on consecutive days were made by a single investigator between 8.30 and 10 AM and averaged.

### Echocardiographic Evaluation

Transthoracic echocardiograms were acquired in anesthetized mice [1.5–2.5% isoflurane v/v (Forene, Abbvie)] using a high frequency ultrasound system (Vevo2100, Visualsonics) while maintaining heart rate at 500 ± 50 beats/min and body temperature between 36–38°C. M-mode images were obtained for left ventricular (LV) function evaluation. Fractional shortening (FS), ejection fraction (EF), LV mass and stroke volume (SV) were calculated using measurements of three consecutive M-mode cycles.

### Western Blot Analyses

Left and right femoral arteries per mouse were pooled and lysed in Laemmli sample buffer (Bio-Rad) containing 5% β-mercaptoethanol. Samples were heat-denatured for 5 min and loaded on Bolt 4–12% or 12% Bis-Tris Gels (Life Technologies). After gel electrophoresis, proteins were transferred to Immobilon-FL membranes (Merck Millipore) according to standard procedures and incubated for 1 h in Odyssey Blocking Buffer (LI-COR Biosciences). Next, membranes were incubated at 4°C overnight with the following primary antibodies: rabbit anti-SQSTM1/p62 (Sigma-Aldrich, P0067) or mouse anti-β-actin (Sigma-Aldrich, A5441). Finally, membranes were incubated with fluorescently labeled secondary antibodies (LI-COR Biosciences, anti-rabbit: IgG926-3221 and anti-mouse: IgG926-68070) to allow IR-detection on an Odyssey SA instrument (LI-COR Biosciences).

### Histology

Femoral arteries and hearts were fixed in 4% formalin for 24 h and paraffin embedded. Transversal sections of the heart were stained with masson’s trichrome staining to determine cardiac fibrosis. Atg7 on femoral artery segments was assessed by immunohistochemistry using anti-Atg7 (Sigma-Aldrich, A2856) monoclonal antibody. Transversal sections of femoral artery segments were stained with hematoxylin-eosin (H&E) to calculate media thickness. Images were acquired with Universal Graph 6.1 software using an Olympus BX40 microscope and quantified with Image J software.

### Statistics

All data are expressed as mean ± SEM with n representing the number of mice. Statistical analyses were performed using Graphpad Prism software (version 8.3.0). Statistical tests are mentioned in the figure legends. *p* < 0.05 was considered as statistically significant. The Shapiro-Wilk test for normal distribution was applied to all data and confirmed normal distribution of the data.

## Results

### Femoral Artery Segments of Atg7^F/F^ SM22α-Cre^+^ Mice Show Features of Autophagy Deficiency

**FIGURE 1:**
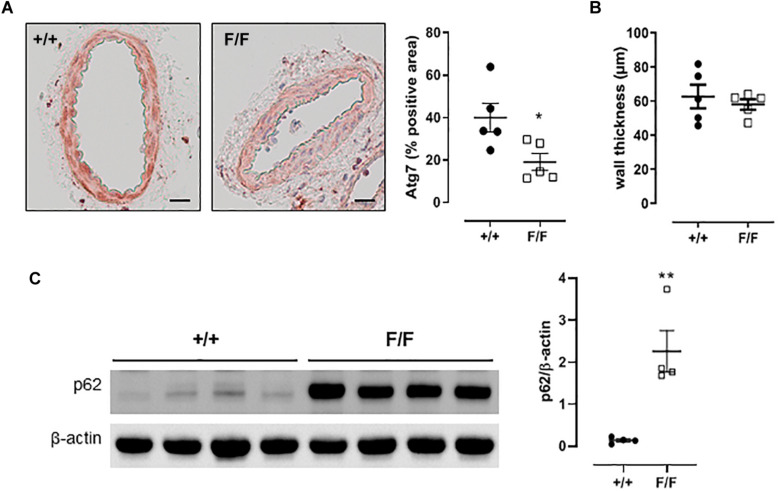
Defective autophagy in VSMCs induces p62 accumulation in the femoral artery. **(A)** Immunostaining for and quantification of Atg7 of femoral artery segments of Atg7^+/+^ SM22α-Cre^+^ (+/+) and Atg7^F/F^ SM22α-Cre^+^ (F/F) mice (*n* = 5). Independent sample *t*-test. *p < 0.05 **p < 0.01. **(B)** Femoral artery media thickness was not affected by autophagy deficiency. **(C)** Western blot analyses of p62 in femoral artery segments of Atg7^+/+^ SM22α-Cre^+^ (+/+) and Atg7^F/F^ SM22α-Cre^+^ (F/F) mice with quantification relatively expressed to β-actin (*n* = 4). Scalebar = 100 μM.

Similar to aortic segments (Grootaert et al., 2015; Michiels et al., 2015), western blot analyses and immunohistochemical analysis of femoral artery segments from Atg7^F/F^ SM22α-Cre^+^ mice revealed the accumulation of p62 and a decrease in Atg7, which are typical features of autophagy deficiency, that were absent in the corresponding Atg7^+/+^ SM22α-Cre^+^ control mice (Figure 1). Femoral artery media thickness was not affected by autophagy deficiency (Figure 1B).

### Defective VSMC Autophagy Enhances Sensitivity to Depolarization-Induced Contractions

**FIGURE 2:**
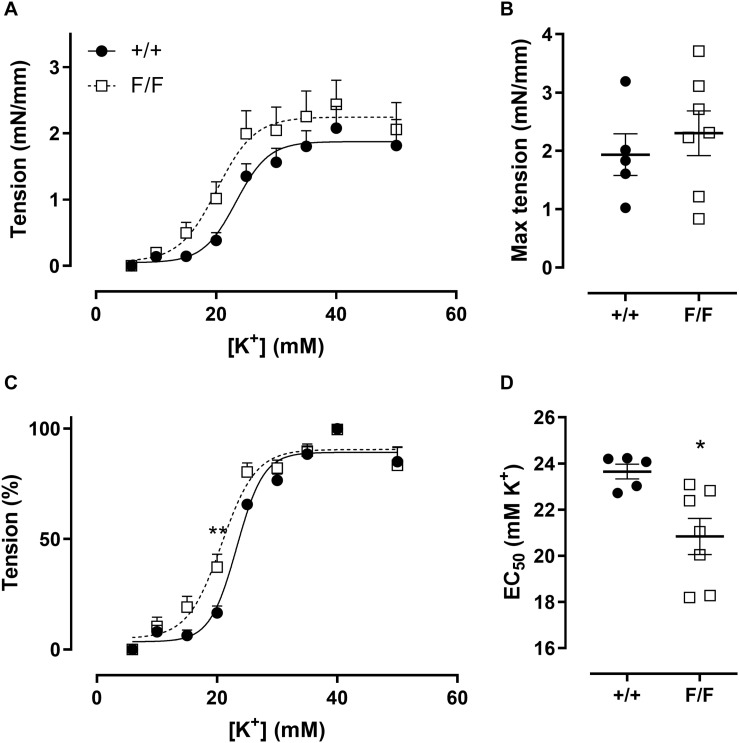
Defective autophagy in VSMCs increases femoral artery sensitivity to depolarization. Absolute **(A)** and relative **(C)** concentration-response curves of contractions elicited by K^+^ in femoral artery segments of Atg7^+/+^ SM22α-Cre^+^ (+/+) and Atg7^F/F^ SM22α-Cre^+^ (F/F) mice and fitted maximal force and EC50 values **(B,D)**. (*n* = 5–7) Two-way ANOVA with sidak *post hoc* test **(A,C)** and independent sample *t*-test **(B,D)**. *p < 0.05, **p < 0.01.

Previous experiments (Michiels et al., 2015) showed that defective VSMC autophagy increased maximally developed force and sensitivity to depolarization in aortic segments. Similar to these observations, force development at different K^+^ concentrations (Figures 2A,C) and respective EC_50_ values (Figure 2D) showed that femoral artery segments of Atg7^F/F^ SM22α-Cre^+^ mice displayed a higher sensitivity to depolarization as compared to Atg7^+/+^ SM22α-Cre^+^ control mice. However, the magnitude of the maximal contractions was not significantly different between both groups (Figure 2B).

### Defective Autophagy in VSMCs Promotes Phasic IP_3_-Mediated Contractions

**FIGURE 3:**
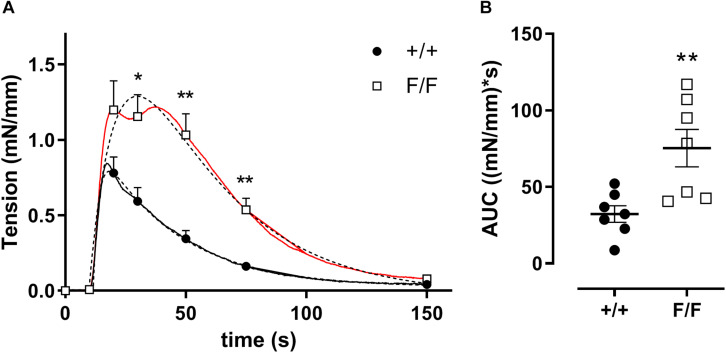
Defective autophagy in VSMCs increases PE mediated phasic contractions in femoral artery segments. IP_3_ mediated transient contractions of Atg7^+/+^ SM22α-Cre^+^ (+/+) and Atg7^F/F^ SM22α-Cre^+^ (F/F) femoral artery segments elicited by 1 μM PE in 0Ca^2+^ conditions **(A)** and area under the curve (AUC) **(B)**. The double exponential fit of the phasic contraction is shown by the dotted lines, whereas the full lines represent the mean data. Some of the data points are shown with SEM. (*n* = 7) Two-way ANOVA with sidak *post hoc* test **(A)** and independent sample *t*-test **(B)**. *p < 0.05, **p < 0.01.

In the absence of extracellular Ca^2+^ (0Ca^2+^), PE causes transient contractions by the release of intracellular Ca^2+^ from the sarcoplasmic reticulum (SR) Ca^2+^ stores via stimulation of inositol 1,4,5-triphosphate (IP_3_) receptors (Kitazawa et al., 1997; Dimopoulos et al., 2007; Fransen et al., 2015). IP_3_-mediated contractions of Atg7^F/F^ SM22α-Cre^+^ femoral artery segments were increased as compared to control femoral artery segments (Figure 3), indicating the presence of larger intracellular Ca^2+^ stores or more Ca^2+^ in the contractile store.

Re-addition of extracellular Ca^2+^ caused tonic contractions, which were the result of NSCC (non-selective cation channel) and VGCC (voltage-gated calcium channel) mediated Ca^2+^ influx (Kitazawa et al., 1997; Dimopoulos et al., 2007; Fransen et al., 2015). These contractions were not different between femoral artery segments of Atg7^F/F^ SM22α-Cre^+^ and Atg7^+/+^ SM22α-Cre^+^ mice (maximal force 1.5 ± 0.2 mN/mm vs 1.9 ± 0.3 mN/mm, *n* = 7, independent sample *t*-test).

### VSMC Sensitivity to NO Is Increased in Atg7^F/F^ SM22α-Cre^+^ Femoral Artery Segments

**FIGURE 4:**
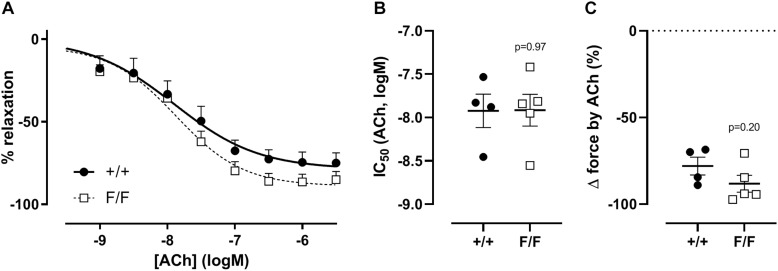
Defective autophagy in VSMCs does not affect endothelium dependent relaxation of femoral artery segments. Relative concentration-response curves of relaxations elicited by acetylcholine (ACh) of Atg7^+/+^ SM22α-Cre^+^ (+/+) and Atg7^F/F^ SM22α-Cre^+^ (F/F) femoral artery segments **(A)**, fitted IC50 **(B)**, and maximal relaxation values **(C)**. (*n* = 3–5) Two-way ANOVA with sidak *post hoc* test **(A)** and independent sample *t*-test **(B,C)**.

In elastic blood vessels such as the aorta, relaxation of the vessel occurs solely via basal or receptor-mediated NO release, whereas in muscular arteries such as the femoral artery, about 70% of the receptor-mediated relaxation occurs via endothelial NO release and about 30% via a non-NO, non-prostanoid factor (Crauwels et al., 2000; Leloup et al., 2015). Because of the absence of basal NO activity in muscular arteries (Leloup et al., 2015), we only investigated the effect of autophagy-deficiency on agonist receptor-mediated relaxation. No statistically significant effect was found in acetylcholine-mediated relaxation in Atg7^F/F^ SM22α-Cre^+^ mice as compared to control mice (Figure 4).

**FIGURE 5:**
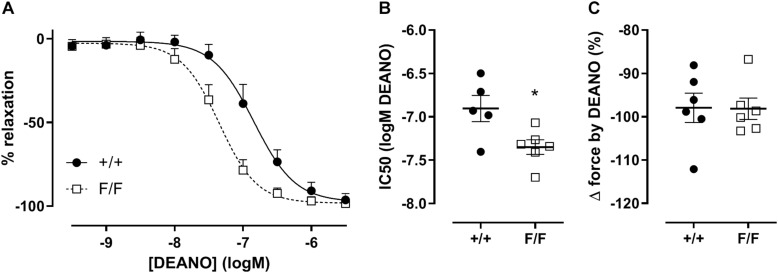
Defective autophagy in VSMCs increases the sensitivity of femoral artery segments to exogenous NO. Relative concentration-response curves of relaxations elicited by DEANO of Atg7^+/+^ SM22α-Cre^+^ (+/+) and Atg7^F/F^ SM22α-Cre^+^ (F/F) femoral artery segments **(A)**, fitted IC50 **(B)** and maximal relaxation values **(C)**. (*n* = 5–6) Two-way ANOVA with sidak *post hoc* test **(A)** and independent sample *t*-test **(B,C)**. *p < 0.05.

The sensitivity of VSMCs to exogenous NO was determined by adding DEANO, an exogenous NO donor, to the organ bath. Although exogenous NO did not cause differences in the magnitude of relaxation, VSMCs from the femoral artery of Atg7^F/F^ SM22α-Cre^+^ were significantly more sensitive to DEANO (Figure 5).

### Angiotensin II-Induced Hypertension Is Absent in Atg7^F/F^ SM22α-Cre^+^ Mice

**FIGURE 6:**
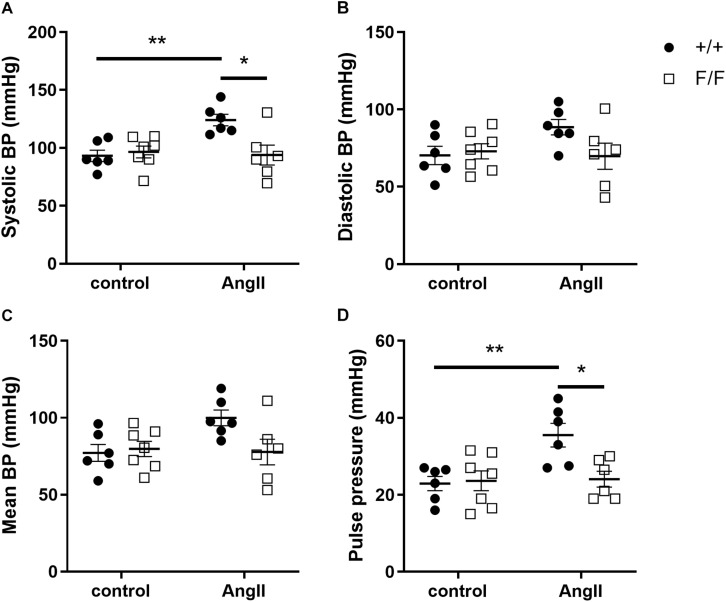
Ang-II treatment increases systolic blood pressure in Atg7^+/+^ SM22α-Cre^+^ but not in Atg7^F/F^ SM22α-Cre^+^ mice. Systolic **(A)**, diastolic **(B)**, mean blood pressure (BP) **(C)**, and pulse pressure **(D)** of Atg7^+/+^ SM22α-Cre^+^ (+/+) and Atg7^F/F^ SM22α-Cre^+^ (F/F) control and AngII-treated mice. (*n* = 6–7) Two-way ANOVA with Sidak *post hoc* test. *p* < 0.05 for genotype factor for systolic BP and pulse pressure. *p < 0.05, **p < 0.01.

Because of differences in large as well as small artery contractility and relaxation, we investigated whether Atg7^F/F^ SM22α-Cre^+^ and Atg7^+/+^ SM22α-Cre^+^ mice differed in their sensitivity to *in vivo* angiotensin II (AngII) treatment. Because longer term autophagy deficiency at the age of 3.5 months was already associated with substantial structural alterations of the aortic vessel wall (data not shown), young animals were used for this purpose. In control conditions, there were no significant differences in systolic, diastolic and mean blood pressure or pulse pressure between Atg7^F/F^ SM22α-Cre^+^ and Atg7^+/+^ SM22α-Cre^+^ mice. One week of AngII treatment raised systolic and pulse pressure in Atg7^+/+^ SM22α-Cre^+^ mice, but not in Atg7^F/F^ SM22α-Cre^+^ mice (Figure 6).

**FIGURE 7:**
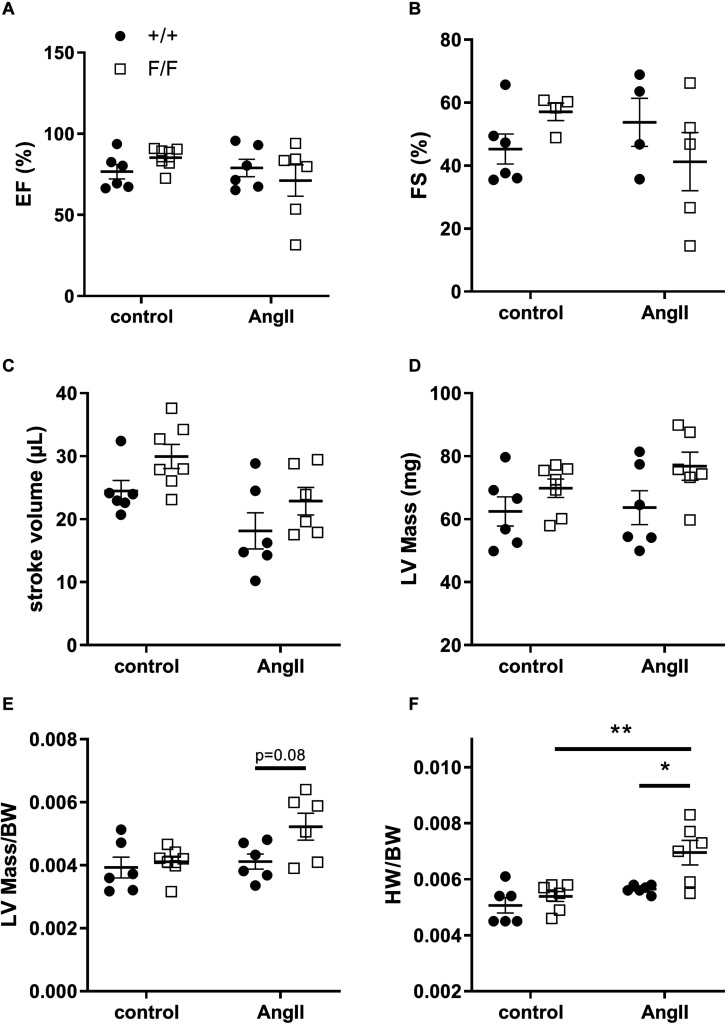
Ang-II treatment causes cardiac hypertrophy in Atg7^F/F^ SM22α-Cre^+^ mice. Ejection fraction **(A)**, fractional shortening **(B)**, stroke volume **(C)**, left ventricular (LV) mass **(D)**, LV mass corrected for body weight (BW) **(E)**, and heart weight (HW) corrected for BW **(F)** of Atg7^+/+^ SM22α-Cre^+^ (+/+) and Atg7^F/F^ SM22α-Cre^+^ (F/F) control and AngII-treated mice. (*n* = 6–7) Two-way ANOVA with Sidak *post hoc* test. *p* < 0.05 for genotype factor for left ventricular mass, stroke volume and LVmass/BW and for angiotensin factor with LVmass/BW. *p* < 0.01 for angiotensin II factor with stroke volume and for genotype factor with HW/BW. *p* < 0.001 for angiotensin II factor with HW/BW. *p < 0.05, **p < 0.01.

To verify whether changes in blood pressure were related to cardiac function, heart parameters were determined. Although no significant differences were found between Atg7^+/+^ SM22α-Cre^+^ and Atg7^F/F^ SM22α-Cre^+^ mice in control conditions, a trend toward better heart performance (EF, FS and stroke volume/cardiac output) and slightly higher cardiac LV mass was observed in Atg7^F/F^ SM22α-Cre^+^ mice. AngII infusion for one week did not affect the different cardiac parameters significantly, but a trend toward a decrease in ejection fraction and fractional shortening as well as a modest increase in left ventricular mass could be observed in the AngII-treated Atg7^F/F^ SM22α-Cre^+^ mice, whereas stroke volume and, in case of similar heart frequency, cardiac output decreased slightly in both animal groups (Figure 7).

**FIGURE 8:**
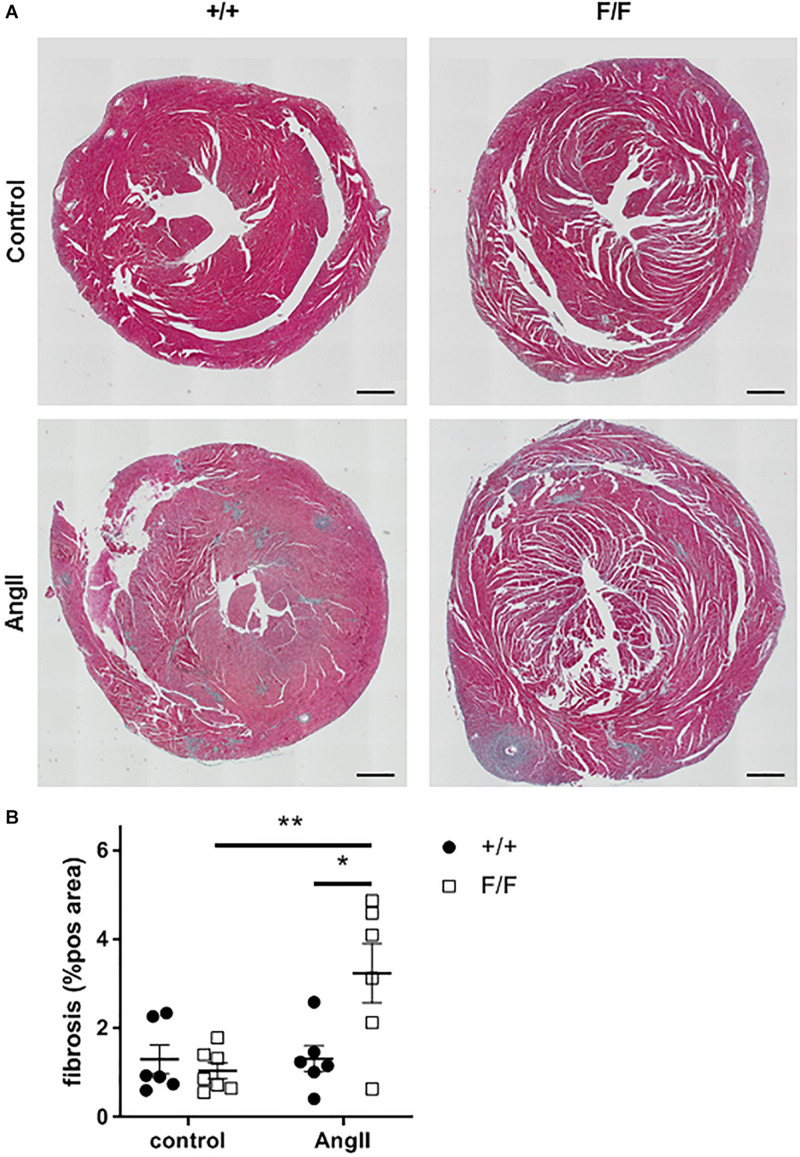
Ang-II treatment increases cardiac fibrosis in Atg7^F/F^ SM22α-Cre^+^ mice. Representative Masson’s trichrome-stained hearts of Atg7^+/+^ SM22α-Cre^+^ (+/+) and Atg7^F/F^ SM22α-Cre^+^ (F/F) control and AngII-treated mice with fibrosis represented as blue stained areas and respective quantification. (*n* = 6–7) Two-way ANOVA with Sidak *post hoc* test *p* < 0.05 for genotype factor and Angiotensin II factor. *p < 0.05 **p < 0.01.

Because of slight variations in body weight (BW), the increase in LV mass was not significant. However, heart weight (HW)/BW ratio revealed a significant increase in AngII treated Atg7^F/F^ SM22α-Cre^+^ mice (Figure 7), thereby strengthening the echocardiography data. Moreover, histological analysis showed that AngII treatment induced cardiac fibrosis in Atg7^F/F^ SM22α-Cre^+^ mice (Figure 8).

## Discussion

The development of hypertension is associated with distinct changes in the vasculature such as endothelial dysfunction, enhanced vascular contractility and arterial remodeling. Indeed, changes in peripheral vascular resistance due to structural and functional alterations in large elastic arteries and small resistance arteries are typical features of hypertension development (Harvey et al., 2015; Brown et al., 2018). Moreover, vascular remodeling, especially in the small arteries is highly correlated with disease severity (Mulvany, 2012). We previously highlighted the importance of VSMC autophagy in the aorta, however, effects on resistance arteries remained essentially unknown. For this reason, in the present study we investigated the effects of defective VSMC autophagy on muscular artery functionality.

Similarly to findings in the aorta (Michiels et al., 2015), VSMC autophagy deficiency sensitized femoral artery contractility. Moreover, femoral artery segments of Atg7^F/F^ SM22α-Cre^+^ mice showed an increase in phasic IP_3_-mediated contractions together with an enhanced sensitivity to depolarization induced contractions. This may be a consequence of an expanded SR with an increased Ca^2+^ storing capacity, as reported previously for the aorta (Michiels et al., 2015). Furthermore, an increased sensitivity to depolarization is also likely to be caused by alterations in the expression levels and sensitivity of voltage gated calcium channels (VGCCs) (Fransen et al., 2016). However, changes in VSMC resting membrane potential, which can also influence VSMC sensitivity to depolarization, cannot be excluded.

Blood pressure strongly depends on the diameter of arterioles, which are major determinants of the resistance to blood flow. Hence, slight changes in the luminal diameter of the smaller vessels have a great impact on blood pressure regulation. Also noteworthy is that alterations in Ca^2+^ influx and cytosolic Ca^2+^ concentrations are involved in the pathogenesis of hypertension. For example, enhanced influx of Ca^2+^ via the L-type Ca^2+^ channel (VGCC) leads to the development of hypertension (Asano and Nomura, 2002; Fransen et al., 2016). Moreover, arterial hypertension itself affects the voltage-sensitivity of VGCCs, which was highly dependent on the vascular bed (Fransen et al., 2016). On the other hand, enhanced expression and sensitivity of IP_3_ receptors also contributes to hypertension since VSMC specific deletion of IP_3_ receptors attenuated AngII-induced hypertension in mice, but did not affect basal blood pressure (Lin et al., 2016). This was in contrast to deletion of the L-type Ca^2+^ channel Cav1.2, which in addition to agonist stimulated hypertension also played a role in basal blood pressure regulation (Moosmang et al., 2003).

Besides changes in VSMC contractility, femoral artery segments of Atg7^F/F^ SM22α-Cre^+^ mice display altered vessel relaxation properties. NO is produced by ECs and induces relaxation of VSMCs directly or indirectly via stimulation of soluble guanylate cyclase (sGC), through removal of cytosolic Ca^2+^ or by decreasing the sensitivity of the contractile apparatus to Ca^2+^ (Akata, 2007). While VSMCs displayed increased sensitivity to exogenous NO, no significant differences in endothelium-dependent relaxation were present. However, because of this increased NO sensitivity, also a rise in VSMC sensitivity to acetylcholine induced relaxation is expected with the same levels of endothelial NO release. Therefore, we speculated that, although it was not directly measured, segments from Atg7^F/F^ SM22α-Cre^+^ mice would have lower agonist-stimulated relaxations. Sensitivity of femoral vessel VSMC to exogenous NO was increased, possibly as a result of enhanced sensitivity of VSMC sGC to NO, which has been observed previously in vessels with reduced endothelial NO release (Fransen et al., 2008). How autophagy deficiency in the VSMCs affects NO release in the endothelium is currently unclear. Nevertheless, it is well known that both cell types in muscular arteries are continuously in cross-talk and are also affected by cardiac output and aortic biomechanical properties (Laurent et al., 2009). Moreover, it has to be noted that, particularly in the femoral artery, other NO-independent mechanisms can contribute to acetylcholine induced VSMC relaxation and these were not investigated in the present study (Crauwels et al., 2000).

Because changes in IP_3_-mediated contractions as well as VSMC NO sensitivity were present in the femoral artery of Atg7^F/F^ SM22α-Cre^+^ mice, we investigated whether this could influence the sensitivity of these animals to AngII-induced hypertension. AngII acts as a potent vasoconstrictor by increasing peripheral resistance via binding to the AngII receptor 1 (AT1), which is predominantly expressed in the resistance vessels. The thoracic aorta on the other hand does not show AngII-induced contractions *ex vivo* due to its low expression of the AT1 receptor (Zhou et al., 2003; Fransen et al., 2016). Because Atg7^F/F^ SM22α-Cre^+^ mice showed increased VSMC sensitivity to NO, we speculated that effects of AngII treatment might blunt the blood pressure increase in these mice. As expected, 7 days of treatment with the hypertensive drug AngII increased systolic blood pressure in control mice, but not in Atg7^F/F^ SM22α-Cre^+^ mice. However, effects of AngII on the heart cannot be excluded. Despite the absence of significant changes in heart parameters, hearts of Atg7^F/F^ SM22α-Cre^+^ mice seemed to perform better in control conditions (a trend toward increased FS and stroke volume), but worse following AngII infusion. In the latter condition, Atg7^F/F^ SM22α-Cre^+^, but not the control Atg7^+/+^ SM22α-Cre^+^ mice had a significantly increased HW/BW ratio in addition to increased cardiac fibrosis, suggesting the occurrence of hypertrophy. Indeed AngII is described to induce heart failure (Wei et al., 2017; Chinnakkannu et al., 2018). Typically, increased blood pressure by AngII treatment causes pressure overload of the heart. This will induce reactive fibrosis and LV hypertrophy in an effort to maintain cardiac output. Eventually, this will fail and cause a decline in cardiac pump function (Pitoulis and Terracciano, 2020). However, in Atg7^+/+^ SM22α-Cre^+^ mice AngII treatment caused hypertension without concomitant cardiac hypertrophy, whereas in Atg7^F/F^ SM22α-Cre^+^ mice cardiac hypertrophy and fibrosis was present without the occurrence of hypertension. Different explanations may account for this. Firstly, unlike the similar biological age of the animals, autophagy deficient animals display accelerated aging (Rubinsztein et al., 2011; Ren and Zhang, 2018; Wu et al., 2019). Hence, there may occur a shift in the time frame of events between both mouse strains: the increase in blood pressure by AngII infusion (measured in Atg7^+/+^ SM22α-Cre^+^ mice, passed over in Atg7^F/F^ SM22α-Cre^+^ animals) precedes cardiac hypertrophy (to occur in Atg7^+/+^ SM22α-Cre^+^ and measured in Atg7^F/F^ SM22α-Cre^+^). This explanation can easily be tested by measuring blood pressure and cardiac parameters at different time points and must be further investigated. Secondly, AngII may affect cardiac function via a direct mechanism independent of increases in blood pressure. Interestingly, this is also described in Balb/CJ mice, where AngII treatment in combination with a high-salt diet did not induce hypertension, but induced cardiac decompensation. This was attributed to the development of acute pulmonary hypertension (Becirovic-Agic et al., 2019). Lastly, heart problems in Atg7^F/F^ SM22α-Cre^+^ mice can develop due to temporal expression of the SM22 gene in cardiomyocytes during embryonic development (Lepore et al., 2005). Indeed, mice with a cardio-specific deletion of Atg5 develop acute pressure overload-induced or β-adrenergic stress-induced cardiac dysfunction, while in baseline conditions no cardiac abnormalities are present (Nakai et al., 2007). In line with these findings, congestive heart failure was described after chronic (12 weeks) infusion of AngII in Atg7^F/F^ SM22α-Cre^+^ mice due to cardiac autophagy deficiency (Ramadan et al., 2018).

In conclusion, VSMC autophagy is not only involved in large elastic artery function but it also plays a pivotal role in the regulation of the contractile and relaxing properties of the smaller muscular arteries. All these effects of autophagy deficiency in the VSMCs point to complex interactions between the heart, elastic and muscular arteries and within each vascular bed between VSMCs and endothelial cells.

## Data Availability Statement

The raw data supporting the conclusions of this article will be made available by the authors, without undue reservation.

## Ethics Statement

The animal study was reviewed and approved by Ethical Committee of the University of Antwerp.

## Author Contributions

DD and PF conception and design of research and interpreted results of experiments. DD, SD, LR, and PF performed the experiments. DD analyzed the data, prepared the figures, and drafted the manuscript. DD, GD, WM, and PF edited and revised the manuscript. DD, SD, LR, GD, WM, and PF approved the final version of the manuscript. All authors contributed to the article and approved the submitted version.

## Conflict of Interest

The authors declare that the research was conducted in the absence of any commercial or financial relationships that could be construed as a potential conflict of interest.

## Supplementary Material

Supplementary Figure 1 Representative example of the experimental protocol applied to the femoral artery segments. **(A)** illustrates the contraction upon increasing extracellular K^+^ and depolarizing the segment. In **(B)**, external Ca^2+^was removed for about 5 min, during which 10 μM PE was applied, leading to a phasic contraction. Then, Ca^2+^ was re-added to the 0 Ca^2+^ solution and after attaining near steady-state tension, ACh was added in a cumulative concentration-response curve. In the final part of the protocol **(C)**, similar PE-induced phasic and tonic contractions were elicited followed by a concentration-response curve for the exogenous NO donor DEANO.
