# Supplementary figures and images for: Defective Autophagy in Vascular Smooth Muscle Cells Alters Vascular Reactivity of the Mouse Femoral Artery
Source: Front Physiol. 2020 Sep 23;11:548943. doi: 10.3389/fphys.2020.548943 (PMC7538838; doi:10.3389/fphys.2020.548943)

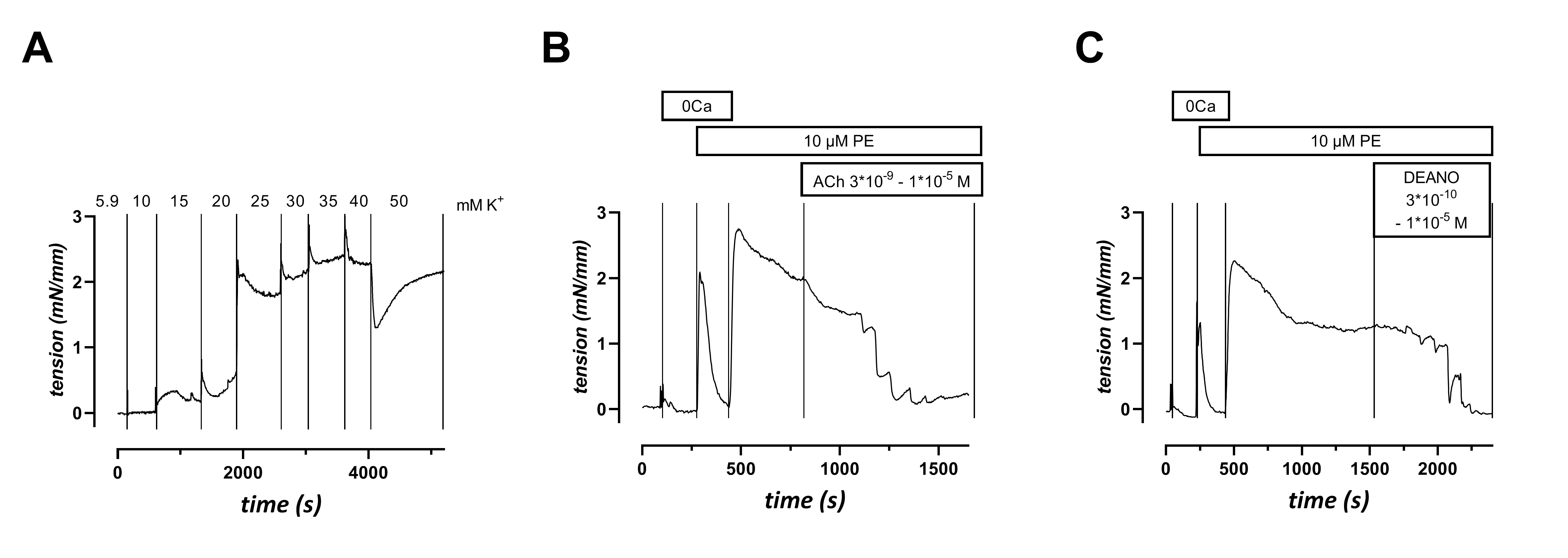

Supplement: Supplementary Figure 1 — Representative example of the experimental protocol applied to the femoral artery segments. (A) illustrates the contraction upon increasing extracellular K+ and depolarizing the segment. In (B), external Ca2+was removed for about 5 min, during which 10 μM PE was applied, leading to a phasic contraction. Then, Ca2+ was re-added to the 0 Ca2+ solution and after attaining near steady-state tension, ACh was added in a cumulative concentration-response curve. In the final part of the protocol (C), similar PE-induced phasic and tonic contractions were elicited followed by a concentration-response curve for the exogenous NO donor DEANO. [file Image_1.TIF]
